# COVID-19 vaccine-related lymph node activation – patterns of uptake on PET-CT

**DOI:** 10.1259/bjrcr.20210040

**Published:** 2021-04-20

**Authors:** Sweni Shah, Thomas Wagner, Malavika Nathan, Teresa Szyszko

**Affiliations:** 1 Department of Nuclear Medicine, Royal Free London NHS Foundation Trust, London, United Kingdom

## Abstract

In a bid to end the current COVID-19 crisis, many countries including UK have begun a mass immunization programme. Immunization can cause transient inflammation thereby causing increased metabolic activity at injection site and hypermetabolic lymph nodes. Various vaccinations and local injections have been known to cause diagnostic dilemma due to false-positive uptake on FDG PET-CT.

In this pictorial case review, we present five cases demonstrating various patterns of uptake including an ipsilateral deltoid muscle, axillary, supraclavicular, and subpectoral lymph nodes post COVID-19 vaccination.

A careful history of COVID-19 vaccination and normal size and morphology of lymph node on unenhanced low-dose CT will aid the diagnosis. All patients undergoing FDG PET-CT will require detailed documentation of the vaccination history including the time interval since vaccination.

Knowledge about these patterns of uptake on PET-CT will ensure accurate interpretation by Nuclear Medicine physicians and radiologists during the current vaccination drive.

## Introduction

Severe acute respiratory syndrome coronavirus 2 (SARS-CoV-2) causes the novel coronavirus disease (COVID-19), a global pandemic.^1^ Many countries including the UK have resorted to ‘national lockdown: stay at home’ measures to curb the spread of disease.^2^ Mass vaccination is considered the main solution to this crisis.

UK vaccination drive began on the 8 December 2020 and vaccines are currently offered to frontline health and social care workers, care home residents and staff, people with chronic conditions including patients on chemotherapy and older adults.^3,4^

Whole-body ^18^ F-2-fluoro-2-deoxy-d-glucose positron emission tomography with low-dose CT (FDG PET/CT) combines functional and anatomical imaging. It has major roles in oncology for staging and post-treatment follow up of many cancers. It is important for the reporting Nuclear Medicine physician and Radiologist to accurately interpret and recognise potential imaging challenges and pitfalls of false-positive FDG avidity.

Vaccinations and injections are known to cause diagnostic dilemma due to false-positive uptake locally on FDG PET-CT.^5^ Several case reports of lymphadenopathy post COVID-19 vaccination have been published recently.^6–10^ To our knowledge, spectrum and patterns of local sites of uptake post COVID-19 vaccination as seen on FDG PET-CT in case review format have not been reported. We aim to present a pictorial review of this phenomenon in COVID-19 vaccinated individuals in order to ensure the imaging community is aware of this pitfall during the current vaccination drive.

## Case 1

**Figure 1.**
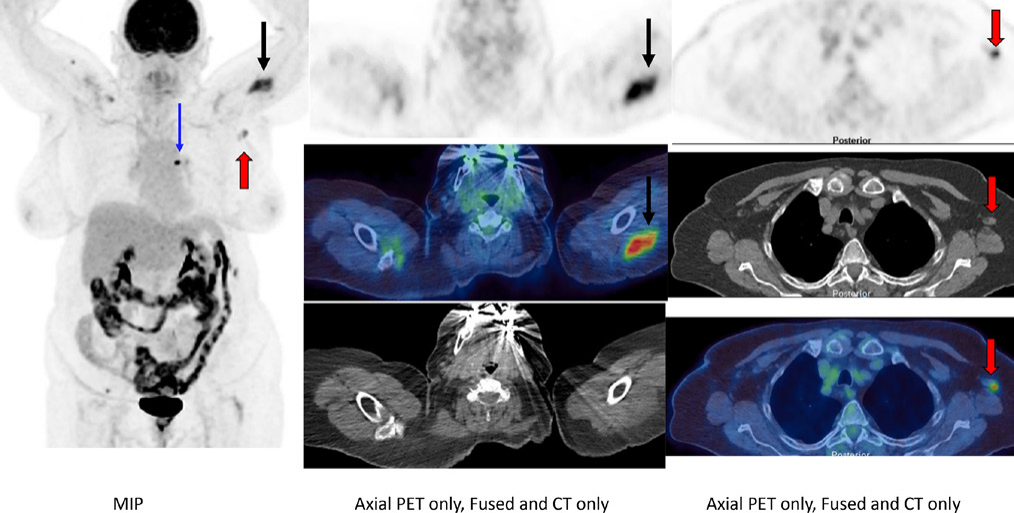
FDG PET-CT (MIP, axial PET only, axial fused and axial CT only) revealed asymmetrical, intense uptake in the left deltoid musculature (black arrows) extending to the cutaneous surface. This was associated with intense uptake in normal sized left axillary lymph nodes (red arrows). These lymph nodes demonstrated a normal fatty hilum (red arrows). Intensely FDG-avid left upper lobe adenocarcinoma is demonstrated on the MIP image (blue arrow).

A 77-year-old female was referred for FDG PET-CT for staging of a biopsy proven left upper lobe non-small cell lung cancer (adenocarcinoma)(Figure 1). Patient had received first dose of COVID-19 vaccine 10 days prior to the FDG PET-CT.

Along with temporal association with history of vaccination, the presence of a normal fatty hilum in normal-sized lymph nodes led to our decision to report vaccine-induced lymphadenopathy. No pathological lymphadenopathy was seen elsewhere.

## Case 2

**Figure 2.**
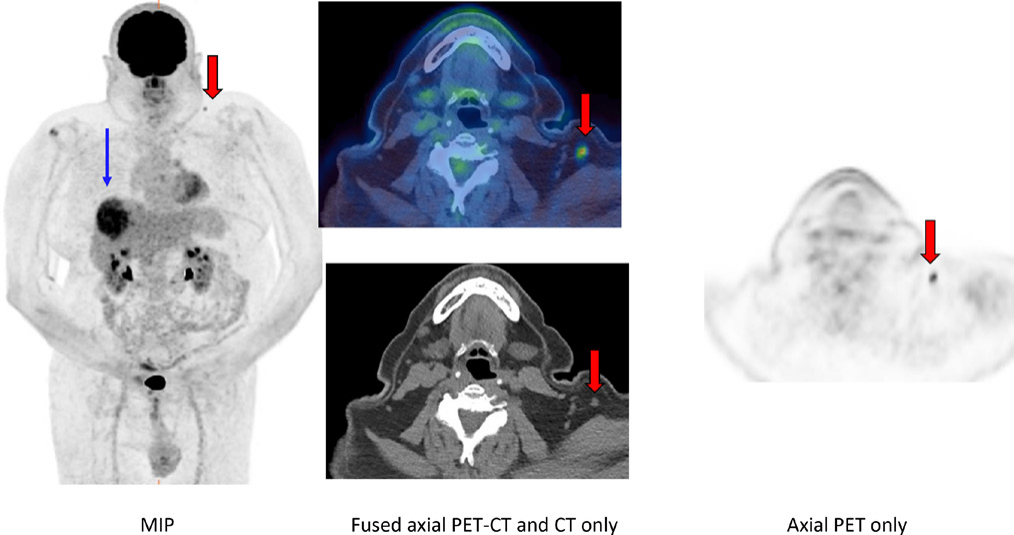
FDG PET-CT (MIP, axial PET only, axial fused and axial CT only) revealed asymmetrical, intense uptake in left supraclavicular normal-sized lymph node (red arrows). The lymph node demonstrated a normal fatty hilum. No asymmetrical uptake was seen in deltoid musculature. MIP images demonstrate intense uptake in primary liver lesion (blue arrow).

A 73-year-old male with a suspicious primary liver lesion underwent FDG PET-CT for staging (Figure 2). Patient had received COVID-19 vaccine three weeks prior to the PET-CT.

No avid lymph nodes were demonstrated elsewhere within the abdomen, pelvis or axillae, hence uptake in the morphologically normal supraclavicular lymph node ipsilateral to the site of vaccination was attributed to reactive lymphadenopathy following vaccination.

## Case 3

**Figure 3.**
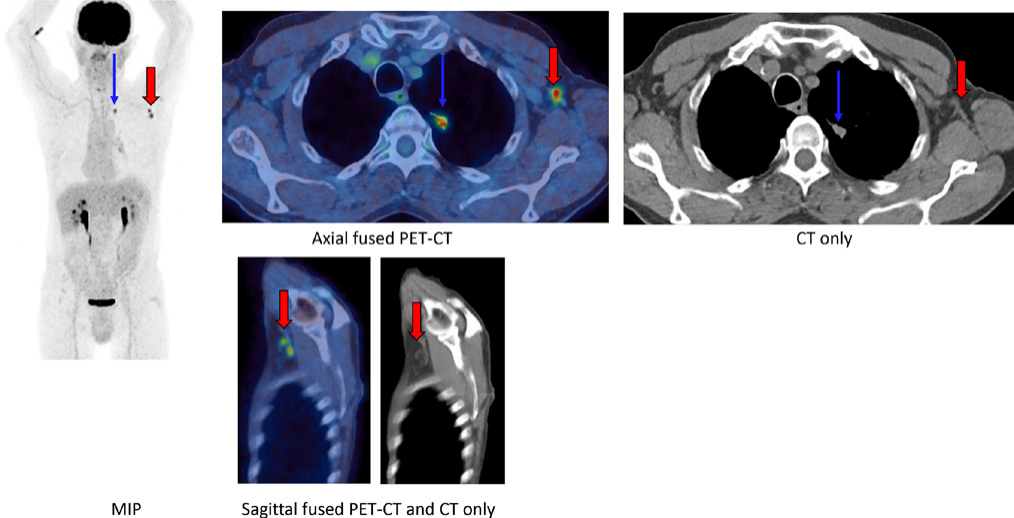
FDG PET-CT (MIP, axial fused PET-CT, axial CT only, sagittal fused PET-CT and sagittal CT only) demonstrated intense asymmetric uptake in two normal-sized left axillary lymph nodes, containing normal fatty hilum (red arrows). No uptake was seen in deltoid musculature. Left upper lobe lung nodule is also demonstrated (blue arrows).

A 72-year-old male underwent a PET-CT for staging of a spiculated left upper lobe nodule (Figure 3). Patient received COVID-19 vaccine on the left side one day prior to the PET-CT

No other avid lymph nodes were demonstrated elsewhere on the PET-CT and uptake within ipsilateral morphologically normal appearing lymph nodes prompted the diagnosis of vaccine-induced adenopathy.

## Case 4

**Figure 4.**
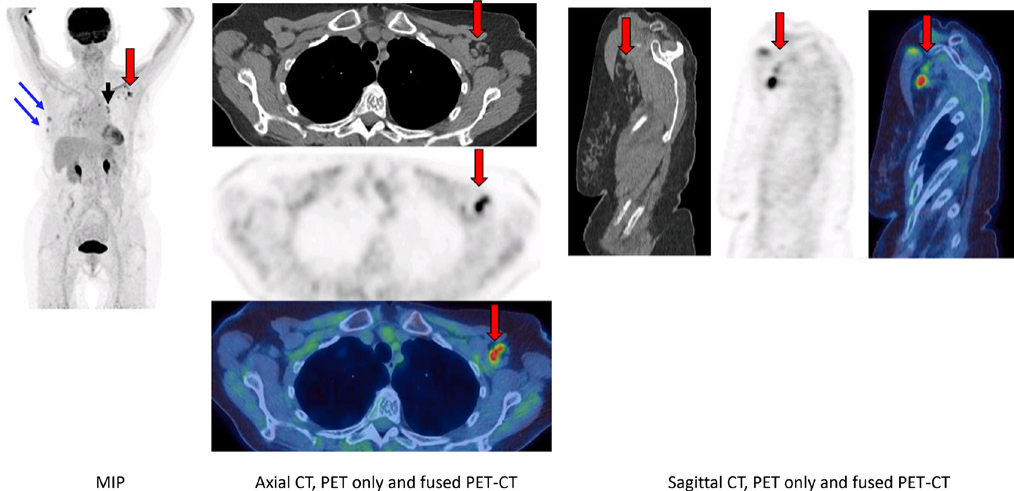
FDG PET-CT (MIP, axial PET only, fused PET-CT, CT only, sagittal PET only, fused PET-CT and CT only) demonstrated unchanged intense uptake in two stable right breast nodules (blue arrows), unchanged intense uptake in bilateral mediastinal and hilar lymph nodes (black arrows). Intense uptake in normal-sized left axillary and subpectoral lymph nodes with normal fatty hilum (red arrows).

An 83-year-old female with primary right-sided breast cancer *in situ* on hormonal and chemotherapy underwent FDG PET-CT for routine three-month follow-up (Figure 4). Patient had COVID-19 vaccine in the left arm 2 weeks prior to PET-CT.

Uptake in all other lymph nodes was stable (when compared to a previous scan) except in supraclavicular and axillary lymph nodes ipsilateral to the injection site, but contralateral to the breast malignancy. Temporal relationship to history of vaccination with morphologically normal but avid lymph nodes prompted the diagnosis of vaccine-related adenopathy.

## Case 5

**Figure 5.**
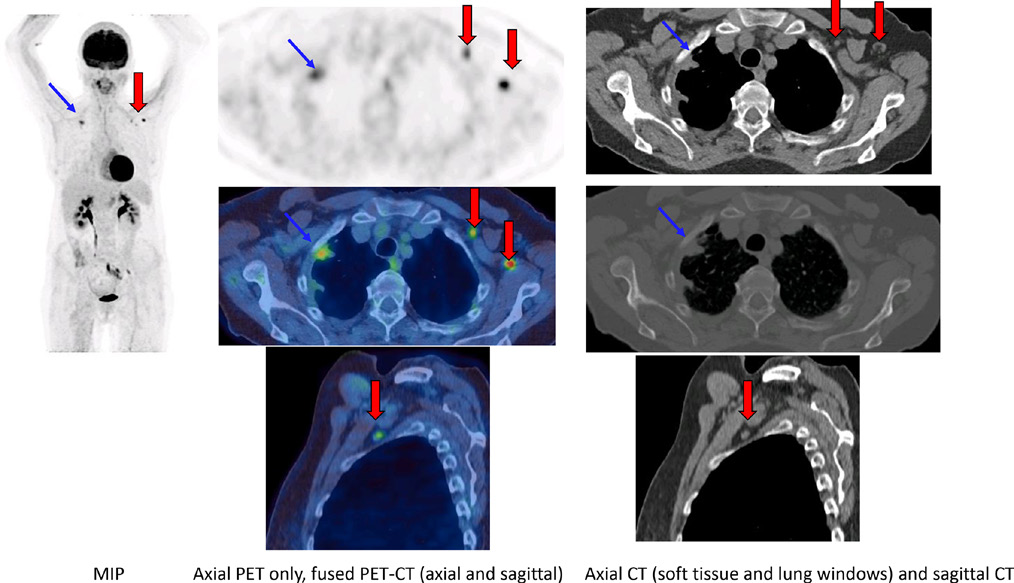
FDG PET-CT (MIP, axial PET only, fused PET-CT, CT only in lung and soft tissue windows, sagittal fused PET-CT and CT only) demonstrated intensely avid uptake in the right apex in keeping with a primary lung malignancy. Intense uptake was seen in normal-sized left axillary and pectoral lymph nodes with a normal fatty hilum.

A 62-year-old female smoker underwent a PET-CT for a suspicious right upper lobe lung nodule found on CT (Figure 5). Patient had a COVID-19 vaccine in the left arm four days prior to the scan.

No other avid lymph nodes were demonstrated elsewhere on the PET-CT and uptake within ipsilateral, morphologically normal appearing lymph nodes prompted the diagnosis of vaccine-induced adenopathy.

## Discussion

Knowledge about potential false-positive results on PET-CT is vital to ensure accurate interpretation during reporting by taking into account the clinical context. Many inflammatory and reactive phenomena are known to cause false-positive avidity.^11^

Vaccination can cause transient inflammation of lymph nodes which demonstrates increased avidity through macrophage accumulation.^12^ Vaccine-related increased metabolic activity at injection site, hypermetabolic lymph nodes, systemic inflammatory response at various sites in different patterns has been reported post-vaccination for the H1N1 pandemic and seasonal influenza vaccines.^13–21^ Standard immunization with diphtheria-tetanus-pertussis with hepatitis B and inactivated polio virus vaccines (DTaP-HepB-IPV) and haemophilus influenzae type b (Hib) with pneumococcal conjugate vaccine (PCV) in paediatric population has also been shown to demonstrate increased uptake at injection site.^22^ Incidence of FDG avidity and reactive nodes post national H1N1 immunization has been reported in up to 30% of patients.^18^ Increased activity has been shown to persist for up to one-month post-influenza vaccination.^14^ Generalised lymph node activation has been reported in immunocompromised (HIV positive) patients post-influenza vaccination. Differences in vaccine formulation and immune response might be the reason for different patterns of response to vaccinations.^23^

Clues towards reactive lymph nodes include history of recent vaccination, normal size and benign morphology of lymph node on unenhanced low-dose CT. Further, comparison with baseline imaging can also be helpful and if there is any doubt clinically, serial imaging, discussion in a multi-disciplinary team setting or tissue sampling (biopsy) can help. Delaying scans for two to four weeks post-vaccination has been suggested; however, we feel this may be practically difficult in oncology patients and may cause unnecessary delays.^24,25^ Routine follow-up scans of potentially low risk or stable cancers can be delayed.

This will avoid errors of staging, patient anxiety, unnecessary investigations (biopsies), overtreatment, surgical resection, change of chemotherapy and radiotherapy treatment plans, excessive follow-up etc.

All our cases were discussed in the relevant multidisciplinary team meeting and a decision was made to perform repeat imaging at three months for follow up as per the institutional protocol.

One major limitation of our case series is that none of our cases underwent biopsy; hence, there was no histological confirmation of the diagnosis and hence no gold standard. In all cases, the diagnosis was made based on the temporal relationship to the vaccination, benign “reactive” morphology on the CT component of the study and consideration of the overall clinical presentation.

It is important to carefully document vaccination history and introduce questions about vaccination including type, time and site of vaccination at the time of performing the scan to ensure these details are available to the reporting NM physician or radiologist at the time of reporting.^13,24^ We have introduced this questionnaire in our department for all patients undergoing PET-CT scanning. In conclusion, we hope this pictorial series will alert the imaging community towards the potential pitfall of reporting nodal uptake in this current vaccination drive and provides a clear illustration of the main patterns of post-vaccine uptake which the reporting physician or radiologist should be aware of.

## Learning points

- All patients undergoing FDG PET-CT should have fully documented COVID-19 vaccination history.
- Normal size and morphology of an avid ipsilateral axillary, supraclavicular or subpectoral lymph nodes on unenhanced low-dose CT shortly after vaccination should alert the reporter towards vaccine-related uptake.
- Knowledge of the patterns of nodal uptake on PET-CT post-vaccination will avoid misinterpretation.
